# Pneumonia in patients with cirrhosis: risk factors associated with mortality and predictive value of prognostic models

**DOI:** 10.1186/s12931-018-0934-5

**Published:** 2018-12-04

**Authors:** Lichen Xu, Shuangwei Ying, Jianhua Hu, Yunyun Wang, Meifang Yang, Tiantian Ge, Chunhong Huang, Qiaomai Xu, Haihong Zhu, Zhi Chen, Weihang Ma

**Affiliations:** 1 State Key Laboratory for Diagnosis and Treatment of Infectious, Collaborative Innovation Center for Diagnosis and Treatment of Infectious Disease, The First Affiliated Hospital, Zhejiang University School of Medicine, 79 Qingchun Road, Hangzhou Zhejiang, 310003 People’s Republic of China; 2 Department of Hematology, Taizhou Hospital of Zhejiang Province, Linhai, Taizhou, China

**Keywords:** Pneumonia, Cirrhosis, Risk factors, Outcome

## Abstract

**Background:**

Cirrhosis always goes with profound immunity compromise, and makes those patients easily be the target of pneumonia. Cirrhotic patients with pneumonia have a dramatically increased mortality. To recognize the risk factors of mortality and to optimize stratification are critical for improving survival rate.

**Methods:**

Two hundred and three cirrhotic patients with pneumonia at a tertiary care hospital were included in this retrospective study. Demographical, clinical and laboratory parameters, severity models and prognosis were recorded. Multivariate Cox regression analysis was used to identify independent predictors of 30-day and 90-day mortality. Area under receiver operating characteristics curves (AUROC) was used to compare the predictive value of different prognostic scoring systems.

**Results:**

Patients with nosocomial acquired or community acquired pneumonia indicated similar prognosis after 30- and 90-day follow-up. However, patients triggered acute-on-chronic liver failure (ACLF) highly increased mortality (46.4% vs 4.5% for 30-day, 69.6% vs 11.2% for 90-day). Age, inappropriate empirical antibiotic therapy (HR: 2.326 *p* = 0.018 for 30-day and HR: 3.126 *p* < 0.001 for 90-day), bacteremia (HR: 3.037 *p* = 0.002 for 30-day and HR: 2.651 *p* = 0.001 for 90-day), white blood cell count (WBC) (HR: 1.452 *p* < 0.001 for 30-day and HR: 1.551 *p* < 0.001 for 90-day) and total bilirubin (HR: 1.059 *p* = 0.002 for 90-day) were independent factors for mortality in current study. Chronic liver failure–sequential organ failure assessment (CLIF-SOFA) displayed highest AUROC (0.89 and 0.90, 95% CI: 0.83–0.95 and 0.85–0.95 for 30-day and 90-day respectively) in current study.

**Conclusions:**

This study found age, bacteremia, WBC, total bilirubin and inappropriate empirical antibiotic therapy were independently associated with increased mortality. Pneumonia triggered ACLF remarkably increased mortality. CLIF-SOFA was more accurate in predicting mortality than other five prognostic models (model for end-stage liver disease (MELD), MELD-Na, quick sequential organ failure assessment (qSOFA), pneumonia severity index (PSI), Child-Turcotte-Pugh (CTP) score).

**Electronic supplementary material:**

The online version of this article (10.1186/s12931-018-0934-5) contains supplementary material, which is available to authorized users.

## Introduction

Cirrhosis is one of the most common causes of mortality worldwide especially in developing countries, with 1-year mortality ranging from 1 to 57% depending on stage [1]. Patients with cirrhosis require frequent medical support, which result in heavy healthcare burden. Cirrhosis not only is a chronic and progressive liver damage, but also involves in a multifactorial immune dysfunction, including uncontrollable cytokines secreting, low phagocytosis of the innate immune and abnormal reaction of T and B cells in pathogen stimulation [2]. Infectious diseases are common in patients with advanced cirrhosis and exert one of most important reasons for mortality. As reported previous, infectious complications increased mortality 4-fold in cirrhotic patients, 30% patients died within 30-day and another 30% died within 1 year after infection [3, 4].

Pneumonia is a common infectious disease in patients with cirrhosis [5, 6]. Importantly, in a infectious disease survey of 4576 cirrhotic patients, pneumonia had a 2.95-fold increase in 30-day mortality, the highest among all infection complications [7] In patients with care unit acquired pneumonia, cirrhosis worsen clinical outcome and increased 28-day mortality as high as 11-fold [8]. Cirrhosis and pneumonia impact each other in pathophysiology. On one hand, cirrhotic host was related to impaired both early and later neutrophil-mediated pulmonary killing of the organisms, making infection uncontrollable [9]; on the other hand, excessive inflammatory factors triggered by pneumonia often lead to rapidly deteriorate liver function and directly damp the anti-bacterial immunity, and further cause multi-organ damage [5, 6].

Although pneumonia exhibits higher mortality in the patient with cirrhosis, few studies focused on the risk factors. To recognize the risk factors of mortality and to optimize stratification is critical for improving survival rate. The purposes of current study were to (1) investigate the epidemiology and outcome of cirrhotic patients with pneumonia; (2) examine independent risk factors for all-cause mortality within 30- and 90-day; (3) compare the value of prognostic models for cirrhotic patients with pneumonia.

## Patients and methods

### Study design and patients

Five thousand seven hundred twenty seven adult cirrhotic patients (≥18 years) from a retrospective cohort referred between January 1, 2013 and January 1, 2015 in the First Affiliated Hospital of Zhejiang University School of Medicine (Hangzhou, China) were screened. Overall 203 cirrhotic patients with pneumonia were included in this study. Cirrhosis was diagnosed by (1) liver biopsy, (2) radiological evidence of liver nodularity and splenomegaly in patients with chronic liver diseases, (3) clinical evidence of signs of portal hypertension or hepatic decompensation (including ascites and hepatic encephalopathy (HE)) [10]. Pneumonia was defined as a new infiltrate focus on chest radiological exam and one or more symptoms as follows: respiratory symptoms (ie cough, chest pain, dyspnea), sign of infection (fever > 38 °C, temperature < 35 °C and/or white blood cell count (WBC) > 12,000/ mm^3^ or < 4000/mm^3^) [10]. The exclusion criteria included: (1) under 18 years; (2) pregnancy; (3) patients with immunosuppression (including patients with leukopenia after chemotherapy, patients with drug-induced immunosuppression as a result of cytotoxic or corticosteroids (defined as>1 mg/kg prednisone for>1 month)); (4) bone marrow or solid-organs transplantation; (5) patients with hepatocellular carcinoma or with other types of carcinoma (6) HIV-infected patients.

### Data collection

The demographic and clinical information were collected: age, sex, smoking, alcohol abuse, etiologies and complications of cirrhosis, co-morbidity; history of operation and pneumonia within 3 months, laboratory parameters and radiographic findings, antibiotic therapy, intensive care unit (ICU) admission, severity models and prognosis. For all patients, the data were collected at diagnosis of pneumonia at admission or up to 5 days after the onset of pneumonia after admission. Community acquired pneumonia (CAP) were those present at admission or developed within the first 48 h after hospitalization. Nosocomial acquired pneumonia (NAP) were those diagnosed after 48 h of admission. Complications of cirrhosis (including ascites, HE, hepatorenal syndrome) were defined in patients according to criteria from the European Association for the Study of the Liver and International Ascites Club [11]. Systemic inflammatory response (SIRS) was diagnosed as at least 2 of the following terms: heart rate > 90 bpm; respiratory rate > 20 bpm; temperature of > 38 °C or < 36 °C; WBC > 12,000/mm^3^ or < 4000/mm^3^ [12]. Bacteremia was defined as positive blood cultures. ACLF was defined as previous description in EASL-CLIF Acute-on-Chronic Liver Failure in Cirrhosis (CANONIC) study [13]. Appropriate empirical antibiotic use was considered as adequate that at least one antibiotic against to the isolated pathogen, according to susceptibility testing, or patients were improvement of clinical signs and laboratory exams of infection after 2–3 days’ empirical antibiotic therapy. Otherwise, the empirical antibiotic therapy was considered inappropriate. Prognostic models used to predict 30-day and 90-day mortality included: PSI, MELD, MELD-Na, CTP score, qSOFA and CLIF-SOFA score. PSI score was calculated as previously described [14]. CTP, MELD and MELD-Na are conventionally used to predicting the outcome of end-stage liver disease. Formula for MELD is: 9.6 × log_e_ (creatinine, mg/dL) + 3.8 × log_e_ (total bilirubin, mg/dL) + 11.2 × log_e_ (INR) + 6.4 × (etiology: 0 for cholestatic or alcoholic cirrhosis, 1 for others) [15]. CTP score composed of ascites, HE, albumin, serum bilirubin and INR [16]. qSOFA includes three aspects: assigning one point for respiratory rate ≥ 22 breaths /min, systolic blood pressure ≤ 100 mmHg, or altered mentation [17]. ACLF-SOFA score was proposed to assess organ failure in ALCF patients by addressing six functional failures (hepatic, renal, cerebral, coagulatory, circulatory and respiratory) [13, 18].

### Statistical analysis

Data were showed as mean ± standard deviations and discrete data were showed as median with the interquartile ranges (IQR). Frequency and percentage were presented for categorical data. Student’s test or Wilcoxon test were used to compare continuous variables in each group. Nominal variables were compared using chi-square test/ Fischer’s exact test. Differences were considered significant at the level of two-sided 0.05. Cox’s proportional hazard regression was used to exam risk factors of time-dependent death. Significant candidate variables (*p* < 0.05) among bivariate analysis and possible variables were entered into a multivariate Cox’s regression by a backward-forward approach. Survival of the patients and subgroups was analyzed using Kaplan–Meier curve and pairwise Log-rank test. Receiver operating curve (ROC) were used to compare the predictive value of different prognostic scoring systems. The Youden index was used to identify the best cut-off point. Statistical analyses were performed using the SPSS software version 20 (IBM Inc., Chicago, IL, USA).

## Result

### Characteristics of population

**Fig. 1:**
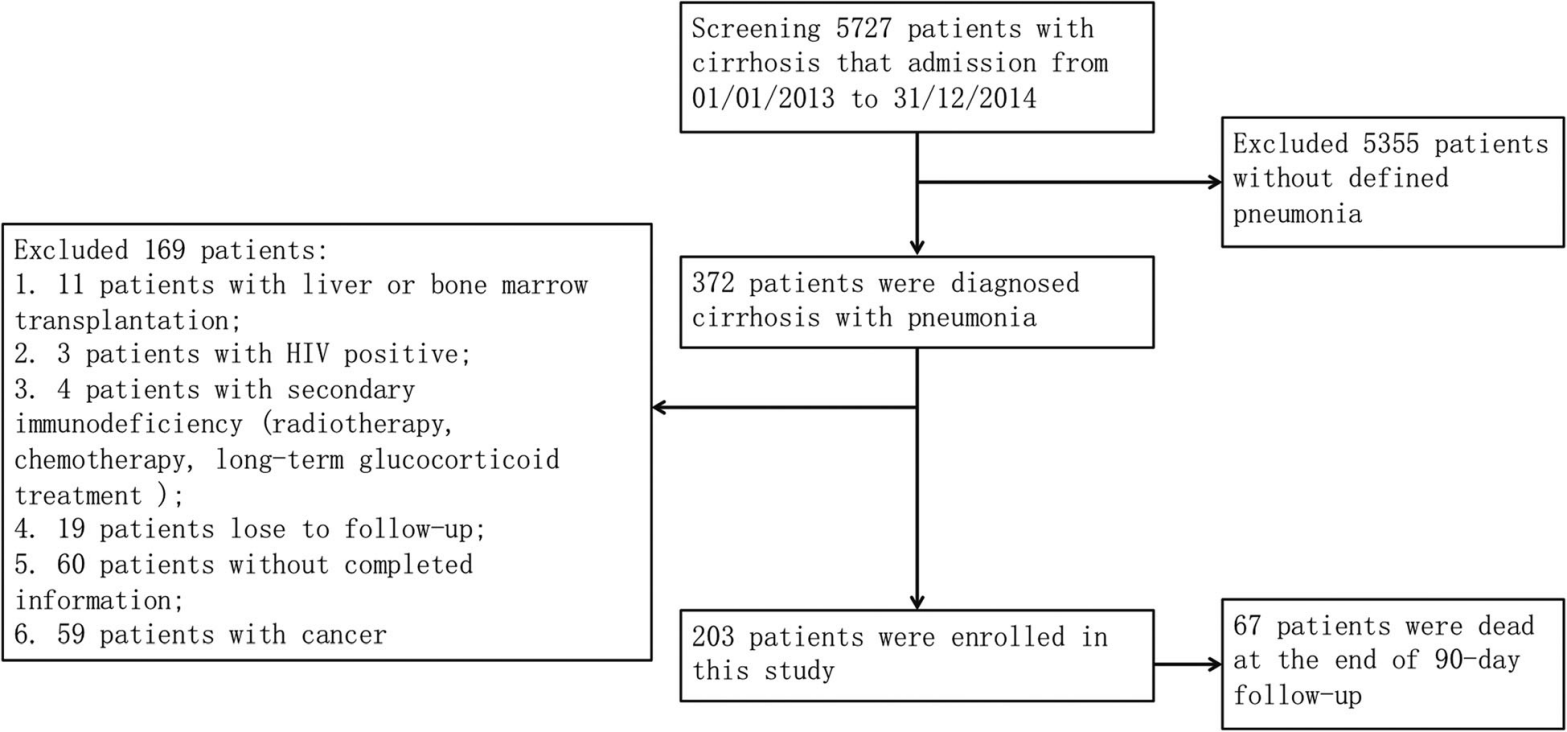
Flow chart of patients screening

**Fig. 2:**
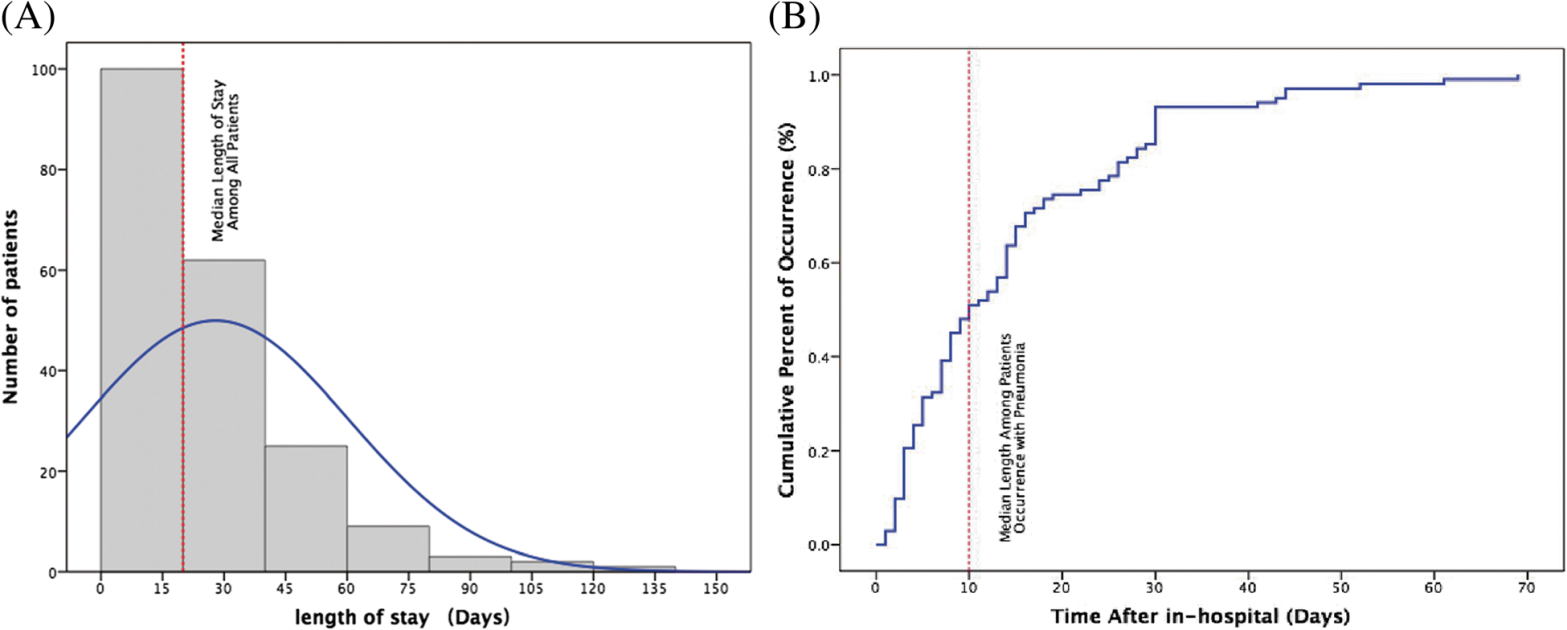
(**a**) the length of stay among all patients; (**b**) the length of pneumonia occurrence among in-hospital cirrhotic patients

During the study, 5727 patients were diagnosed cirrhosis, 372 patients with cirrhosis and pneumonia. 169 patients were excluded as listed: 11 patients with liver or bone marrow transplantation; 3 patients with HIV positive; 4 patients with drug-induced immunosuppression; 19 patients lost to follow-up; 60 patients without completed information; 59 patients with cancer. 203 patients were enrolled in this study, 67 patients (31.0%) were non-survival and 136 patients (69.0%) were survival at the end of 90-day follow-up (Fig. 1). The median length of stay among cirrhotic patients with pneumonia was 20 days (Fig. 2a). Among nosocomial acquired patients, the median length of stay was 10 days before occurrence of pneumonia (Fig. 2b). Baseline characteristics and prognosis of the overall study cohort were depicted in Table 1. Survival patients did not differ significantly from non-survival in relation to the causes of cirrhosis (virus 52.1% vs. 55.6%, alcohol 17.3% vs. 14.3%, others 35.0% vs. 36.5%). As shown in Table 1, there were no significant differences between the survival and non-survival groups in sex, past medical history, smoking, antibiotic pre-treatment, and co-morbidities. In non-survival group, patients showed higher frequency of decompensated complication, ascites (90.5% vs. 72.9% *p* = 0.005), hepatorenal syndrome (30.2% vs. 5.7% *p* < 0.001) and high grade (III-IV) HE (15.9% vs. 0.7% *p* < 0.001). Of note, bacteremia was more frequently observed in non-survived group (14.3% vs. 3.6% *P* = 0.013). Significant differences were also observed in ICU admission (33.3% vs. 7.9% *p* < 0.001), SIRS (79.4% vs. 50.7% *p* < 0.001), and appropriate empirical antibiotic use (15.9% vs. 72.1% *p* < 0.001).

**Table 1:** Comparison of clinical characteristics between survivors and non-survivors patients for 90-day follow-up

Variables	Total, *n* = 203(100%)	Survivor, *n* = 140(69.0%)	Non-survivors, *n* = 63(31%)	*P* value
Demographics data				
Age(years), M ± SD	57.1 ± 13.7	57.1 ± 13.8	57.0 ± 13.6	0.952
Sex m/f (% male)	138(68.0%)	95(67.9%)	43(68.3%)	0.955
Current smoking *n* (%)	74(36.5%)	54(38.6%)	20(31.7%)	0.431
Alcohol abuse *n* (%)	67(33.0%)	50(35.7%)	17(27.0%)	0.260
Antibiotic therapy within the 3 months *n* (%)	42(20.4%)	34(24.3%)	8(12.7%)	0.064
History of operation within the 3 months *n* (%)	16(7.9%)	13(9.3%)	3(4.8%)	0.400
History of pneumonia within the 3 months *n* (%)	18(8.9%)	14(10.0%)	4(6.3%)	0.594
Co-morbidity				
^a^Chronic respiratory disease *n* (%)	16(7.9%)	12(8.6%)	4(6.3%)	0.780
^b^Chronic cardiovascular disease *n* (%)	11 (5,.4%)	7 (5.0%)	4 (6.3%)	0.742
^c^Chronic renal disease *n* (%)	8 (4.0%)	5 (3.6%)	3 (4.8%)	0.706
Diabetes mellitus *n* (%)	31 (15.3%)	21 (15.0%)	10 (15.9%)	0.827
^d^Neurological disease *n* (%)	17 (8.4%)	10 (11.1%)	7 (11.1%)	0.414
Etiology of cirrhosis				
Virus *n* (%)	108 (53.2%)	73 (52.1%)	35 (55.6%)	0.852
Alcohol *n* (%)	33 (16.3%)	24 (17.3%)	9 (14.3%)	
Others *n* (%)	72 (35.5%)	49 (35.0%)	23 (36.5%)	
Complications of cirrhosis				
Ascites *n* (%),	159 (78.3%)	102 (72.9%)	57 (90.5%)	0.005
Variceal bleeding *n* (%)	57 (28.1%)	36 (25.7%)	21 (33.3%)	0.312
SBP *n* (%)	29 (14.3%)	17 (12.1%)	12 (19.0%)	0.200
Hepatorenal syndrome *n* (%)	27 (13.3%)	8 (5.7%)	19 (30.2%)	<0.001
HE grade III/IV *n* (%)	11 (5.4%)	1 (0.7%)	10 (15.9%)	<0.001
Laboratory and radiographic findings				
WBC 10^9^ cells/L (IQR)	9.2 (5.4–14.0)	8.1 (4.7–11.9)	13.8 (8.6–21.6)	<0.001
Platelet count, 10^9^ platelets/L(IQR)	60.0 (32.0–93.0)	69.5 (41.3–103.0)	43.0 (21.0–68.0)	<0.001
C-reactive protein level, mg/dL (IQR)	27.9 (12.3–66.8)	27.0 (10.6–66.3)	28.7 (15.6–68.8)	0.314
Creatinine, μmol/L (IQR)	76 (59.0–120.0)	68.0 (55.0–90.8)	118 (76.0–212.0)	<0.001
Albumin, g/dL (M ± SD)	25.7 ± 4.9	26.0 ± 5.0	25.0 ± 4.8	0.180
Total Bilirubin, mg/dL (IQR)	63.5 (24.8–239.8)	38.0 (21.3–104.0)	273 (71.2–479.5)	<0.001
INR (IQR)	1.54 (1.27–2.23)	1.36 (1.20–1.70)	2.62 (1.93–3.38)	<0.001
Multilobar infiltration *n* (%)	155 (77.5%)	102 (72.9%)	53 (88.3%)	0.017
Pleural effusion *n* (%)	153 (76.1%)	102 (72.9%)	51 (83.6%)	0.109
Bacteremia n (%)	14 (6.9%)	5 (3.6%)	9 (14.3%)	0.013
SIRS n (%)	121 (59.6%)	71 (50.7%)	50 (79.4%)	<0.001
Appropriate empirical antibiotic use *n* (%)	111 (54.7%)	101 (72.1%)	10 (15.9%)	<0.001
ICU addmission *n* (%)	32 (15.8%)	11 (7.9%)	21 (33.3%)	<0.001
Severity score				
PSI score (IQR)	107 (88–140)	99 (87–115)	164 (124–196)	<0.001
MELD (M ± SD)	17.6 ± 12.6	12.2 ± 8.7	29.7 ± 11.3	<0.001
MELD-Na (M ± SD)	21.4 ± 18.8	15.5 ± 11.6	34.5 ± 15.0	<0.001
Child-Pugh C grade, n (%)	84 (41.4%)	36 (25.7%)	48 (76.2%)	<0.001
qSOFA (IQR)	0 (0–1)	0 (0–1)	1 (1–2)	**<0.001**
CLIF-SOFA (M ± SD)	7.4 ± 4.3	5.4 ± 2.9	11.5 ± 3.9	**<0.001**

^a^Chronic respiratory disease was defined as previous diagnosis of chronic obstructive pulmonary disease (COPD) and/or asthma

^b^Chronic cardiovascular disease was defined as previous diagnosis of coronary artery disease (myocardial infarction) and/or congestive heart failure

^c^Chronic renal disease was defined as previous diagnosis of chronic renal failure (including patients undergoing dialysis)

^d^Neurological disease was defined as previous diagnosis of cerebral hemorrhage and/or infarction

### Comparison the characteristics of CAP and NAP

According to the diagnosis points, patients were divided into CAP group and NAP group and the characteristics for each group were listed in Additional file 1: Table S1. There were no differences in age, sex, smoking and alcohol abuse between CAP and NAP. However, patients with NAP showed higher rates with decompensated cirrhosis, ascites (72.1% vs. 85.9% *p* = 0.025) and variceal bleeding (21.6% vs. 35.9% *p* = 0.029). Patients with CAP showed less frequently admitted to the ICU (10.8% vs. 22% *P* = 0.049). Patients with NAP and CAP indicated similar prognosis after 30- and 90-day follow-up.

### ACLF occurrence was associated with undesirable prognosis

**Fig. 3:**
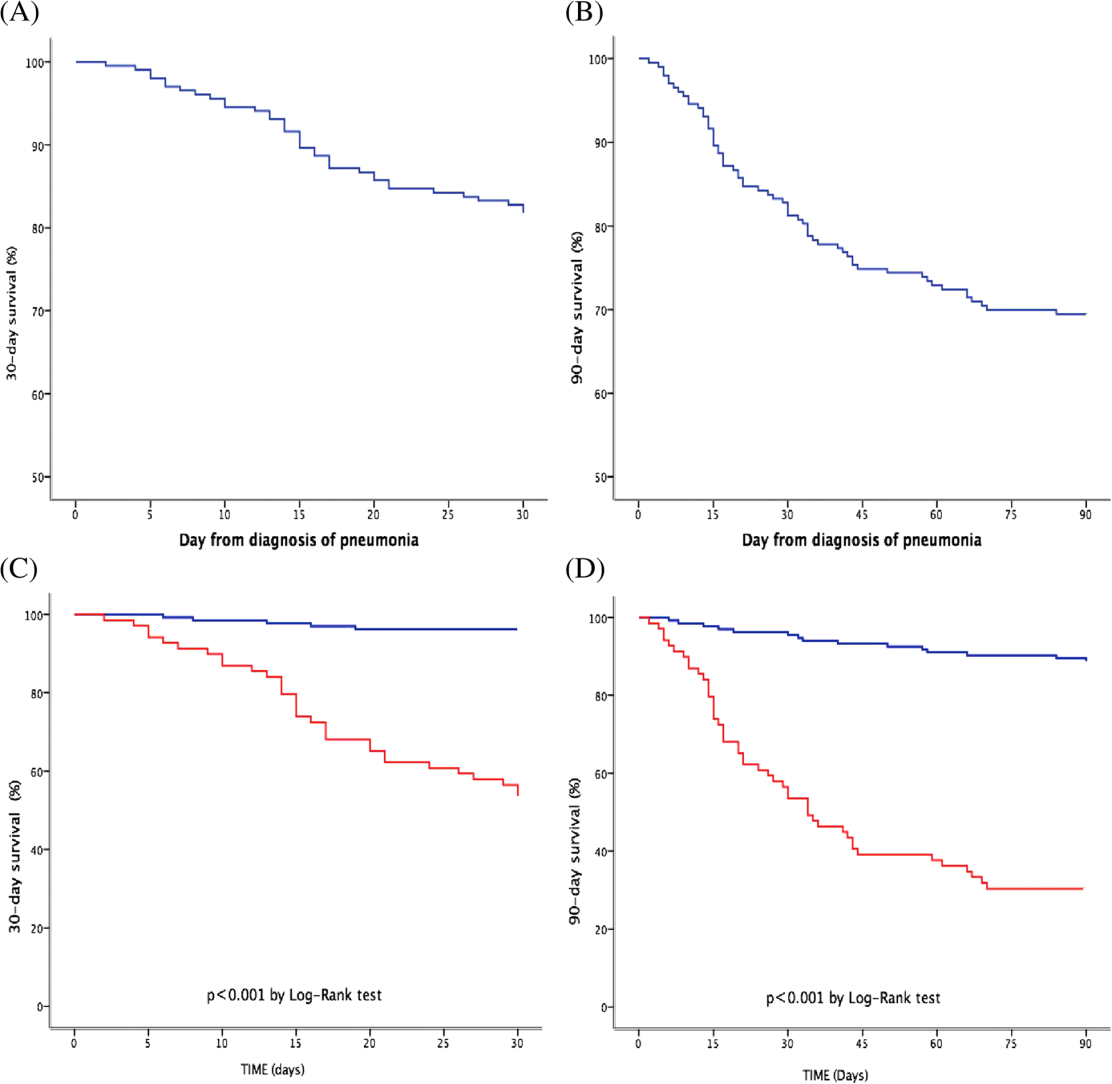
Kaplan-Meier curve depicting 30-day (**a**) and 90-day (**b**) mortality; Kaplan-Meier curve depicting survival of patients with and without acute-on-chronic liver failure within 30-day (**c**) and 90-day (**d**) mortality

Cirrhotic patients with pneumonia were divided into ACLF and non-ACLF group and shown in Table 2. Sixty-nine patients (34.0%) were diagnosed ACLF according to EASL-CLIF definition. 30-day and 90-day mortality rate were 46.4 and 69.6% respectively. In contrast, patients without ACLF exhibited significantly lower mortality rate, 4.5 and 11.2% for 30-day (Fig. 3a and c) and 90-day (Fig. 3b and d) respectively. Patients with ACLF had no statistical differences in age to those without ACLF (55.4 ± 13.7 vs. 57.9 ± 13.7). The frequency of ascites (87.0% vs. 73.9% *p* = 0.03), hepatorenal syndrome (33.3% vs. 3.0% *p* < 0.001) and HE (31.9% vs. 6.7% *p* < 0.001) at diagnosis of pneumonia were significantly higher in ACLF group. Analytical parameters showed a worse profile in ACLF group (lower platelet count and higher INR, bilirubin and creatinine).

**Table 2:** Baseline characteristics and laboratory results of patients with ACLF and without ACLF

Variables	ACLF *n* = 69 (34.0%)	NO ACLF *n* = 134 (66.0%)	*P* value
Demographics data			
Age(years), M ± SD	55.4 ± 13.7	57.9 ± 13.7	0.23
Sex m/f (% male)	52 (75.4%)	86 (64.2%)	0.12
Complications of cirrhosis			
Ascites *n* (%)	60 (87.0%)	99 (73.9%)	0.03
Variceal bleeding *n* (%)	23 (33.3%)	29 (21.6%)	0.09
Spontaneous bacterial peritonitis *n* (%)	11 (15.9%)	18 (13.4%)	0.67
Hepatorenal syndrome *n* (%)	23 (33.3%)	4 (3.0%)	<0.001
HE *n* (%)	22 (31.9%)	9 (6.7%)	<0.001
Laboratory and radiographic findings			
White blood cell count, 10^9^ cells/L	13.8 (8.65–21.95)	8.1 (4.7–11.6)	<0.001
Platelet count, 10^9^ platelets/L (IQR)	47 (22–75.5)	68.5 (40.8–101.5)	0.001
C-reactive protein level, mg/dL (IQR)	28.8 (14.7–72.0)	26.9 (11.6–65.5)	0.38
Creatinine, μmol/L (IQR)	154.0 (83.5–256.5)	66.5 (53.8–80.0)	<0.001
Albumin, g/dL (M ± SD)	24.8 ± 4.8	26.2 ± 4.9	0.06
Total Bilirubin, mg/dL (IQR)	252.0 (62.5–470.5)	38.0 (22.0–99.5)	<0.001
INR (IQR)	2.37 (1.62–3.38)	1.39 (1.22–1.73)	<0.001
Multi-lobar infiltration *n* (%)	55 (80.9%)	100 (75.8%)	0.48
SIRS n (%)	53 (76.8%)	68 (50.7%)	<0.001
ICU addmission *n* (%)	24 (35.3%)	8 (6.0%)	<0.001
EASL-CLIF definition ACLF			
ACLF-1 (%)	13 (18.8%)		
ACLF-2 (%)	28 (40.6%)		
ACLF-3 (%)	28 (40.6%)		
PSI score (IQR)	159 (117–195)	96 (86–115)	<0.001
Mortality			
30-day mortality	32 (46.4%)	6 (4.5%)	<0.001
90-day mortality	48 (69.6%)	15 (11.2%)	<0.001

### Factors associated with 30- and 90-day mortality

Cirrhosis patients with pneumonia in present cohort had 30- and 90-day mortality rate 18.7 and 31.0%, respectively. The factors that associated with 30- and 90-day mortality were listed in Additional file 2: Table S2. The factors may related to mortality were employed into univariate Cox regression analysis. After univariate analysis, the factors with statistical significance were entered in multivariate Cox regression analysis. Age was an important factor that linked to undesirable prognosis in pneumonia as previous description, therefore, it was also took into multivariate calculation. In baseline parameters model, several clinical manifestations were analyzed the contribution to 30- and 90-day mortality. As was showed in Table 3, inappropriate empirical antibiotic therapy was critical for predicting 30 days (HR = 2.326 95%CI: 1.158–4.673 *p* = 0.018) and 90 days (HR = 3.126 95%CI: 1.726–5.662 *p* < 0.001) mortality. Besides, bacteremia was also related to higher mortality (HR = 2.651 95%CI: 1.488–4.724 *p* = 0.001 for 90-day and HR = 3.037 95%CI: 1.515–6.090 *p* = 0.002 for 30-day). Laboratory exam results that related to prognosis were analyzed in model 2. Multivariate analysis showed that the following parameters were related to mortality for 90-day: age (HR = 1.026 95%CI: 1.006–1.047 *p* = 0.013), WBC (HR = 1.551 95%CI: 1.358–1.771 *p* < 0.001), total bilirubin (HR = 1.059 95%CI:1.021–1.098 p = 0.002). However, 30-day mortality was only associated with WBC (HR = 1.452 95%CI: 1.257–1.676 *p* < 0.001). It was worth noting that age was an important factor in 90-day mortality no matter in baseline parameters model (HR = 1.020 95%CI: 1.001–1.041 *p* = 0.043) or in laboratory exam model (HR = 1.026 95%CI: 1.006–1.047 *p* = 0.013). The conventional severity models for pneumonia and liver disease mortality prediction were assessed in the last model (severity scores model).

**Table 3:** Risk factors associated with transplant-free 30-day and 90-day mortality in cirrhotic patients with pneumonia

Variables	90-day				30-day			
	Univariate		Multivariate		Univariate		Multivariate	
	HR (95% CI)	*P* value	HR (95% CI)	*P* value	HR (95% CI)	*P* value	HR (95% CI)	*P* value
	Model 1: Baseline parameters				Model 1: Baseline parameters			
Age	1.000 (0.982–1.018)	0.998	1.020 (1.001–1.041)	0.043	1.004 (0.981–1.027)	0.761		
Multilobar infiltration	2.474 (1.125–5.443)	0.024			1.954 (0.760–5.027)	0.164		
Bacteremia	2.88 (1.419–5.843)	0.003	2.651 (1.488–4.724)	0.001	2.293 (0.894–5.877)	0.084	3.037 (1.515–6.090)	0.002
Inappropriate empirical antibiotic use	8.894 (4.516–17.516)	<0.001	3.126 (1.726–5.662)	<0.001	7.645 (3.194–18.295)	<0.001	2.326 (1.158–4.673)	0.018
SIRS	3.061 (1.662–5.637)	<0.001			2.788 (1.278–6.084)	0.01		
Ascites	2.931 (1.264–6.800)	0.012			1.918 (0.749–4.912)	0.175		
Hepatorenal syndrome	3.847 (2.238–6.614)	<0.001			3.013 (1.494–6.076)	0.002		
Hepatic encephalopathy	3.534 (2.073–6.023)	<0.001			3.416 (1.746–6.683)	<0.001		
	Model 2: Laboratory exam				Model 2: Laboratory exam			
Age	1.000 (0.982–1.018)	0.998	1.026 (1.006–1.047)	0.013	1.004 (0.981–1.027)	0.761	1.024 (0.997–1.051)	0.082
WBC (10^9^ /L)	1.070 (1.048–1.091)	<0.001	1.551 (1.358–1.771)	<0.001	1.066 (1.040–1.092)	<0.001	1.452 (1.257–1.676)	<0.001
Platelet count (10^9^/L)	0.986 (0.979–0.994)	<0.001			0.989 (0.980–0.998)	0.012		
INR	1.273 (1.190–1.362)	<0.001			1.242 (1.163–1.326)	<0.001		
Total Bilirubin (mg/dL)	1.003 (1.001–1.004)	<0.001	1.059 (1.021–1.098)	0.002	1.003 (1.001–1.004)	0.004		
Hemoglobin (g/L)	0.985 (0.975–0.996)	0.006			0.991 (0.978–1.004)	0.159		
HCT	0.938 (0.903–0.976)	0.001			0.955 (0.912–1.000)	0.052		
Creatinine (μmol/L)	1.003 (1.001–1.004)	<0.001			1.003 (1.001–1.004)	0.004		
Glucose	1.075 (1.046–1.105)	<0.001			1.060 (1.019–1.102)	0.004		
	Model 3: Severity scores				Model 3: Severity scores			
CLIF-SOFA score	1.319 (1.250–1.393)	<0.001			1.296 (1.213–1.386)	<0.001		
Child-Pugh C grade	7.024 (3.193–15.450)	<0.001			5.198 (2.025–13.344)	0.001		
MELD	1.096 (1.076–1.117)	<0.001			1.086 (1.061–1.112)	<0.001		
MELD-Na	1.066 (1.050–1.083)	<0.001			1.062 (1.041–1.083)	<0.001		
PSI scores	1.023 (1.018–1.028)	<0.001			1.024 (1.017–1.031)	<0.001		
qSOFA≥2	6.248 (3.752–10.402)	<0.001			8.654 (4.546–16.474)	<0.001		

### Value of prognostic models in cirrhotic patients with pneumonia

**Fig. 4:**
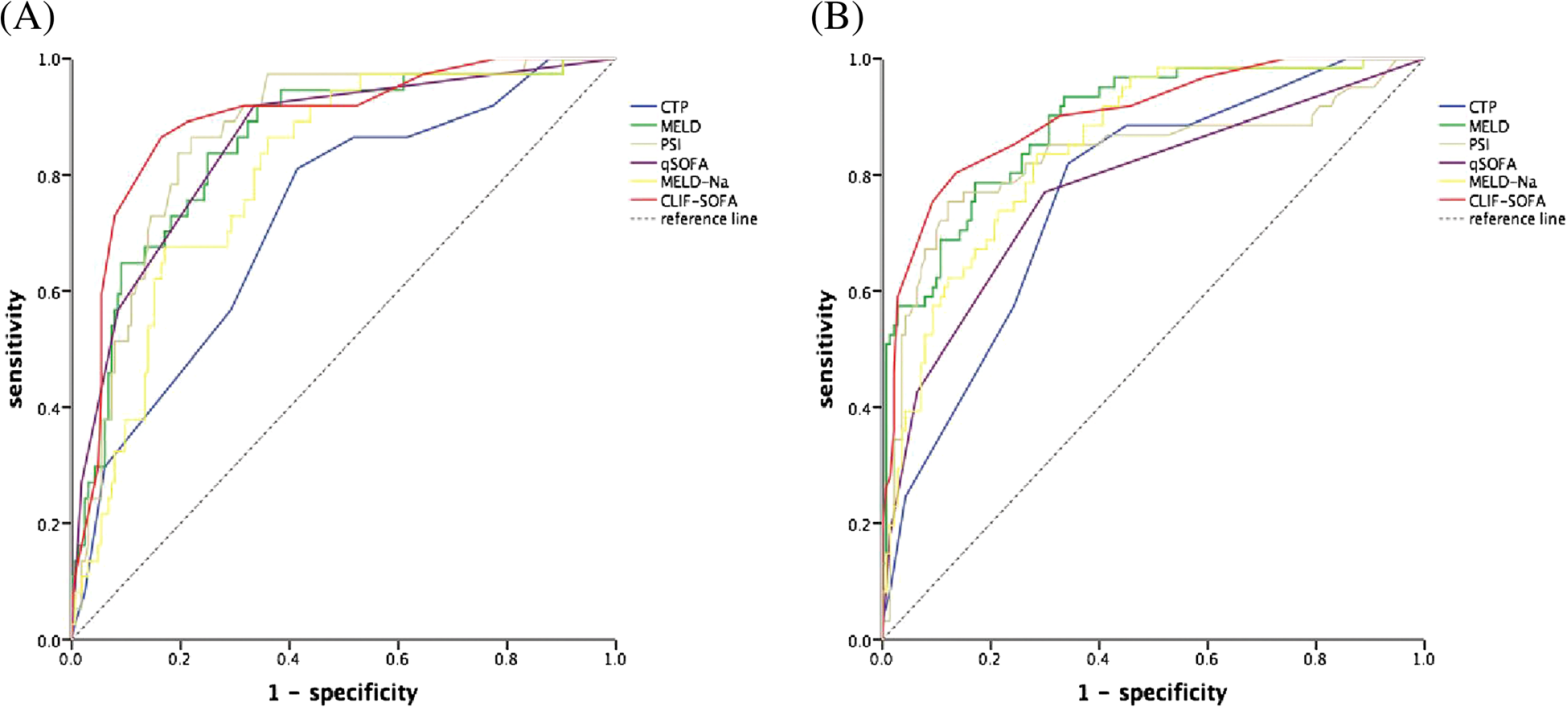
Receiver operating curves (ROC) of prognostic models in predicting 30-day (**a**), 90-day (**b**) mortality in cirrhotic patients with pneumonia

Six prognostic models were tested for predicting 30-day and 90-day mortality in cirrhotic patients with pneumonia. (Fig. 4 and Table 4) Among those prognosis models, AUROC of CLIF-SOFA in predicting 30-day (AUROC: 0.89, 95%CI: 0.83–0.95) and 90-day (AUROC: 0.90, 95%CI: 0.85–0.95) mortality at onset of pneumonia were higher than those of five other predicting models. Using Youden index, the best cut-off point for CLIF-SOFA was 9.5 for 30-day mortality (sensitivity: 0.803 and specificity: 0.864) and 8.5 for 90-day (sensitivity: 0.803 and specificity: 0.864) mortality.

**Table 4:** Performance of six prognostic scoring systems for predicting mortality of cirrhosis patients with pneumonia

Time Point	Prognosis Model	AUROC (95% CI)	Youden Index	Sensitivity	Specificity
30-day	CLIF-SOFA	0.890 (0.830–0.950)	0.700	0.865	0.835
	MELD	0.853 (0.787–0.920)	0.557	0.649	0.909
	MELD-Na	0.801 (0.730–0.872)	0.505	0.676	0.829
	qSOFA	0.854 (0.786–0.922)	0.584	0.919	0.665
	PSI	0.867 (0.808–0.926)	0.645	0.865	0.780
	CTP	0.726 (0.638–0.814)	0.396	0.811	0.585
90-day	CLIF-SOFA	0.900 (0.852–0.947)	0.668	0.803	0.864
	MELD	0.889 (0.840–0.937)	0.615	0.787	0.829
	MELD-Na	0.849 (0.794–0.904)	0.523	0.787	0.736
	qSOFA	0.777 (0.703–0.852)	0.470	0.770	0.700
	PSI	0.831 (0.758–0.903)	0.633	0.754	0.879
	CTP	0.768 (0.700–0.837)	0.477	0.820	0.657

## Discussion

To the best of our knowledge, this retrospective study represented the largest contemporary epidemiological survey of pneumonia in cirrhotic patients. In present study, we evaluated the epidemiology, clinical characteristics, prognosis and the value of severity predicting score in cirrhotic patients with pneumonia. We found 1) mortality of pneumonia in in-hospital cirrhotic patients were as high as 31%, the median length before NAP occurrence after admission and the median length of hospital stay were 10 days and 20 days, respectively; 2) cirrhotic patients with NAP or CAP showed no difference in 30-day and 90-day mortality; 3) inadequate empirical antibiotic therapy, bacteremia and WBC were independent factors for increasing 30-day and 90-day mortality in current study, while age and total bilirubin were independent factors for 90-day mortality but not for 30-day; 4) it dramatically increased 30-day and 90-day mortality that cirrhotic patients with pneumonia coincided with ACLF than those who not; 5) CLIF-SOFA was more accurate in predicting 30-day and 90-day mortality for cirrhotic patients with pneumonia.

Infection is a common complication in cirrhotic patients, especially in patients with advanced cirrhosis. Pneumonia was reported as the third common infection complication in cirrhosis [6]. Few studies have discussed the epidemiology and characteristics of pneumonia in cirrhotic patients. However, in clinical situation, pneumonia in cirrhosis dramatically increases mortality. To recognize the risk factors for mortality is critical for improving survival rate. Cirrhotic patients often show profound immunodeficiency and systematic inflammation, which make them easily get infectious diseases [19]. As reported previously, bacterial infections occurred in 25–35% of admitted patients with cirrhosis [4]. In current study, 5727 adult patients who admitted with cirrhosis were included. The occurrence rate of pneumonia was 6.5%, lower than prior reports [7, 20, 21]. That was because cirrhotic patients in all stages were investigated in the study, but other studies only investigated patients with end-stage cirrhosis. However, the mortality rate was striking similarly. Our results showed that mortality were as high as 18.7 and 33.0% in 30- and 90-day follow-up, respectively. In previous studies, cirrhotic patients with pneumonia may associate with higher mortality than with other site infections, nevertheless, the mechanism was indefinite [7]. Innate immunity deficiency in cirrhotic patients always leads to impairment of lung bacteria clearance and uncontrollable pro-inflammatory cytokines production [22]. In addition, systemic inflammation has been shown to favor serious complications such as HE, variceal bleeding and acute-on-chronic liver failure [23–25]. However, it still requires further investigation that whether inflammatory response in cirrhotic patients with pneumonia is stronger than with other infections and leads to worse prognosis.

CAP and NAP in cirrhosis patients were compared in clinical features in our study. Although patients with CAP presented slight severity in liver function, there were no differences in 30- and 90-day mortality. That may result from early use of antibiotics in community. However, patients who developed ACLF dramatically affected 30- and 90-day mortality. In the CANONIC trial, bacteria infection was a predominant extra-hepatic event triggering ACLF [13]. Pneumonia the second main event trigger ACLF. NO surprisingly, pneumonia was a main precipitating event causing ACLF. As reported by Marcus M. Mücke et al. [26], SBP (32.4%) was the most common cause of ACLF, followed by pneumonia (25.4%). It was worth to note that the occurrence rate of pneumonia was significantly lower than SBP in previous reports. (13.6% vs 24.1% [21], 9.1% vs 24.9% [27]) In other words, pneumonia may much easier to cause ACLF than SBP, despite of comparatively lower incidence rate. In our research, 34% patients complicated with pneumonia triggered ACLF and the mortality rate after 90-day follow-up was 69.6%, which was similar to the previous reports [26, 28].

Severe respiratory infection often coincides with hypoxia. A continuous supply of oxygen is required to secure a sufficient energetic supply for cellular activity. In hypoxia context, low oxygen supply induces higher-level oxygen free radical and lower–level cellular activity, consequently, aggravating tissue damage and reducing bacteria and toxicity clearance in liver. The further homeostasis disturbances induce multi-organ failure. However, the higher rate of pneumonia in ACLF triggering is still unknown.

Because of high mortality of pneumonia in cirrhotic patients, the risk factors for mortality were discussed in our study. In general, age, the severity of infection and liver function were important in influence of 90-day prognosis. Interestingly, age may not influent short-term mortality, but important for 90-day mortality in both baseline parameters model and laboratory exam model. Appropriate empirical antibiotic use was crucial in improving survival of patients. Our study found that 3-fold (30 days) and 2.3-fold (90 days) increase mortality for patients who were prescribed inappropriate empirical antibiotic therapy, which was similar to cirrhotic patients with blood stream infection [29, 30]. Compared to other prognostic models for severity of pneumonia and end-stage liver disease, CLIF-SOFA preformed better in predicting outcome. The advantage of CLIF-SOFA is apparent. CLIF-SOFA scale system takes circulation and respiratory function into consideration, which are also important factors for evaluation of severity of pneumonia. Compared to PSI index, the scale system for assess severity of pneumonia, CLIF-SOFA is more accurate in asssessing liver function.

Our study had some limitations. First of all, our study was a single-center cohort and retrospect design. Considered low number of patients, our observations should be confirmed in larger sample studies. Secondly, our study excluded cirrhosis patients with cancer, immunosuppression and organ transplantation, besides, patients who lost in follow-up and without sufficient information were also excluded in current study. Therefore, exclusions may result in statistic bias and inexact conclusion. At last, we failed to demonstrate a significant relationship between the cirrhosis with pneumonia and high mortality. Although evidences showed cirrhosis with pneumonia may imply higher risk of ACLF and higher mortality, the potential mechanisms were not clear.

Nevertheless, our investigation provided first comprehensive study of prognosis of cirrhotic patients with pneumonia. Cirrhosis patients who complicated with pneumonia were at high risks of undesirable prognosis. Age, WBC, bacteremia and total bilirubin were significant in affecting outcomes, while appropriate empirical antibiotic therapy was important in improving survival. CLIF-SOFA model was superior to other scoring system in predicting 30-day and 90-day mortality.

## Additional files

Additional file 1: **Table S1.** Comparison of clinical feature between community acquired and nosocomial acquired pneumonia in cirrhotic patients. (DOCX 20 kb)

Additional file 2: **Table S2.** Predictors of 90-day and 30-day mortality in the univariate analysis in cirrhotic patients with pneumonia. (DOC 64 kb)

## Abbreviations

ACLF: Acute on chronic liver failure
AUROC: Area under the receiver operating characteristic curves
CAP: Community acquired pneumonia
CI: Confidence interval
CLIF-SOFA: Chronic liver failure–sequential organ failure assessment
CTP: Child-Turcotte-Pugh
HE: Hepatic encephalopathy
ICU: Intensive care unit
INR: International normalized rate
IQR: Interquartile ranges
MELD: Model for end-stage liver disease
NAP: Nosocomial acquired pneumonia
OR: Odds rate
PSI: Pneumonia severity index
qSOFA: Quick sequential organ failure assessment
SBP: Spontaneous bacteria peritonitis
SIRS: Systemic inflammatory response syndrome
UTI: Urinary tract infection
WBC: White blood cell count
