# Supplementary material for: Pneumonia in patients with cirrhosis: risk factors associated with mortality and predictive value of prognostic models
Source: Respir Res. 2018 Dec 4;19:242. doi: 10.1186/s12931-018-0934-5 (PMC6280505; doi:10.1186/s12931-018-0934-5)
Supplement: Supplementary file 1 — Table S1. Comparison of clinical feature between community acquired and nosocomial acquired pneumonia in cirrhotic patients. (DOCX 20 kb) [file 12931_2018_934_MOESM1_ESM.docx]

| Variables | CAP, n=111 (54.6%) | NAP, n=92 (45.3%) | *P* value |
| --- | --- | --- | --- |
| **Demographics data** |  |  |  |
| Age(years), M±SD | 56.6±13.5 | 57.7±14.0 | 0.572 |
| Sex m/f (% male) | 74 (66.7%) | 64 (69.6%) | 0.763 |
| Current smoking *n* (%) | 39 (35.1%) | 35 (38.0%) | 0.770 |
| Alcohol abuse *n* (%) | 35 (31.5%) | 32 (34.8%) | 0.655 |
| **Complications of cirrhosis** |  |  |  |
| Ascites *n* (%), | 80 (72.1%) | 79 (85.9%) | **0.025** |
| Variceal bleeding *n* (%) | 24 (21.6%) | 33 (35.9%) | **0.029** |
| SBP *n* (%) | 15 (13.5%) | 14 (15.2%) | 0.841 |
| Hepatorenal syndrome *n* (%) | 11 (9.9%) | 16 (17.4%) | 0.147 |
| HE grade Ⅲ/Ⅳ *n* (%) | 9 (8.1%) | 2 (2.2%) | 0.116 |
| **Laboratory and radiographic findings** |  |  |  |
| WBC, 10^9^ cells/L | 10.6±8.4 | 12.3±8.7 | 0.163 |
| Platelet count, 10^9^ platelets/l (IQR) | 66 (39-101) | 53 (29-83) | **0.049** |
| C-reactive protein level, mg/dl (IQR) | 22.1 (9.1-66.3) | 36.7 (16.3-69.4) | **0.028** |
| Creatinine, μmol/L (IQR) | 75 (57-108) | 78 (59-134) | 0.378 |
| Albumin, g/dL (M±SD) | 25.74±5.4 | 25.70±4.3 | 0.947 |
| Total Bilirubin, mg/dl (IQR) | 38 (22-129) | 94 (38-398) | **＜0.001** |
| INR ( (IQR)) | 1.44 (1.22-2.12) | 1.64 (1.31-2.39) | **0.033** |
| Multi-lobar infiltration *n* (%) | 82 (52.9%) | 73 (47.1%) | 0.309 |
| **Bacteraemia n (%)** | 6 (5.4%) | 8 (8.7%) | 0.520 |
| **SIRS n (%)** | 65 (58.6%) | 56 (60.9%) | 0.849 |
| **ICU addmission *n* (%)** | 12 (10.8%) | 20 (22%) | **0.049** |
| **Severity score** |  |  |  |
| PSI score (IQR) | 107 (89-133) | 109 (88-155) | 0.565 |
| CLIF-SOFA (M±SD) | 6.72±4.44 | 8.02±4.05 | **0.032** |
| **Mortality** |  |  |  |
| 90-day mortality | 29 (26.1%) | 34 (37.0%) | 0.127 |
| 30-day mortality | 22 (19.8%) | 16 (17.4%) | 0.720 |

Additional file 1: Table 1. Comparison of clinical feature between community acquired and nosocomial acquired pneumonia in cirrhotic patients
