# Supplementary material for: Pneumonia in patients with cirrhosis: risk factors associated with mortality and predictive value of prognostic models
Source: Respir Res. 2018 Dec 4;19:242. doi: 10.1186/s12931-018-0934-5 (PMC6280505; doi:10.1186/s12931-018-0934-5)
Supplement: Supplementary file 2 — Table S2. Predictors of 90-day and 30-day mortality in the univariate analysis in cirrhotic patients with pneumonia. (DOC 64 kb) [file 12931_2018_934_MOESM2_ESM.doc]

Additional file 2: Table S2. Predictors of 90-day and 30-day mortality in the univariate analysis in cirrhotic patients with pneumonia

| Variables | 90-day |  | 30-day |  |
| --- | --- | --- | --- | --- |
|  | HR (95% CI) | *P* value | HR (95% CI) | *P* value |
| Age | 1.000 (0.982-1.018) | 0.998 | 1.004 (0.981-1.027) | 0.761 |
| Male | 1.027 (0.604-1.746) | 0.921 | 1.023 (0.516-2.027) | 0.948 |
| History of pneumonia within the 3 months | 0.640 (0.232-1.761) | 0.387 | 0.553 (0.133-2.296) | 0.415 |
| Splenectomy |  |  |  |  |
| Virus-related cirrhosis | 1.165 (0.709-1.915) | 0.547 | 1.415 (0.738-2.712) | 0.296 |
| Multi-lobar infiltration | **2.474 (1.125-5.443)** | **0.024** | 1.954 (0.760-5.027) | 0.164 |
| Bacteremia | **2.88 (1.419-5.843)** | **0.003** | 2.293 (0.894-5.877) | 0.084 |
| Corticosteroid therapy | **1.775 (1.273-2.476)** | **0.001** | **1.576 (1.041-2.384)** | **0.031** |
| Inappropriate empirical antibiotic use | **8.894 (4.516-17.516)** | **＜0.001** | **7.645 (3.194-18.295)** | **＜0.001** |
| C-reactive protein levels (mg/L) | 1.003 (0.999-1.008) | 0.166 | 1.003 (0.997-1.009) | 0.260 |
| SIRS | **3.061 (1.662-5.637)** | **＜0.001** | **2.788 (1.278-6.084)** | **0.01** |
| Ascites | **2.931 (1.264-6.800)** | **0.012** | 1.918 (0.749-4.912) | 0.175 |
| Hepatorenal syndrome | **3.847 (2.238-6.614)** | **＜0.001** | **3.013 (1.494-6.076)** | **0.002** |
| Variceal bleeding | 1.611 (0.954-2.721) | 0.075 | 1.757 (0.909-3.397) | 0.094 |
| Spontaneous bacterial peritonitis | 1.474 (0.786-2.765) | 0.227 | 1.133 (0.474-2.709) | 0.780 |
| Hepatic encephalopathy | **3.534 (2.073-6.023)** | **＜0.001** | **3.416 (1.746-6.683)** | **＜0.001** |
| **Cirrhotic complication** | **3.805 (1.382-10.477)** | **0.01** | 2.036 (0.722-5.737) | 0.179 |
| White blood cell count (10^9^ /L) | **1.070 (1.048-1.091)** | **＜0.001** | **1.066 (1.040-1.092)** | **＜0.001** |
| Platelet count (10^9^/L) | **0.986 (0.979-0.994)** | **＜0.001** | **0.989 (0.980-0.998)** | **0.012** |
| INR | **1.273 (1.190-1.362)** | **＜0.001** | **1.242 (1.163-1.326)** | **＜0.001** |
| PT | 1.000 (0.999-1.001) | 0.750 | 1.000 (0.999-1.001) | 0.796 |
| Albumin (g/dL) | 0.967 (0.919-1.019) | 0.208 | 0.996 (0.933-1.064) | 0.910 |
| Total Bilirubin (mg/dl) | **1.003 (1.001-1.004)** | **＜0.001** | **1.003 (1.001-1.004)** | **0.004** |
| Hemoglobin (g/L) | **0.985 (0.975-0.996)** | **0.006** | 0.991 (0.978-1.004) | 0.159 |
| HCT | **0.938 (0.903-0.976)** | **0.001** | 0.955 (0.912-1.000) | 0.052 |
| Creatinine (μmol/) | **1.003 (1.001-1.004)** | **＜0.001** | **1.003 (1.001-1.004)** | **0.004** |
| Glucose | **1.075 (1.046-1.105)** | **＜0.001** | **1.060 (1.019-1.102)** | **0.004** |
| Serum Na (mmol/L) | 1.008 (0.971-1.046) | 0.679 | 1.028 (0.986-1.071) | 0.197 |
| PaO2 < 60 mmHg | **4.491 (2.731-7.385)** | **＜0.001** | **4.745 (2.506-8.983)** | **＜0.001** |
| ACLF-SOFA score | **1.319 (1.250-1.393)** | **＜0.001** | **1.296 (1.213-1.386)** | **＜0.001** |
| Child-Pugh C grade | **7.024 (3.193-15.450)** | **＜0.001** | **5.198 (2.025-13.344)** | **0.001** |
| MELD | **1.096 (1.076-1.117)** | **＜0.001** | **1.086 (1.061-1.112)** | **＜0.001** |
| MELD-Na | **1.066 (1.050-1.083)** | **＜0.001** | **1.062 (1.041-1.083)** | **＜0.001** |
| PSI 4-5 grade | **3.352 (1.596-7.040)** | **＜0.001** | **16.584 (2.275-120.891)** | **0.006** |
| qSOFA≥2 | **6.248 (3.752-10.402)** | **＜0.001** | **8.654 (4.546-16.474)** | **＜0.001** |
